# Unusual presentation of sarcoidosis: solitary intracranial mass lesion mimicking an intracranial neoplasm: a case report

**DOI:** 10.11604/pamj.2014.18.236.1409

**Published:** 2014-07-22

**Authors:** Amen Ghozzi, Heifa Azouz, Inès Chelly, Haifa Nfoussi, Hafedh Jemal, Nidhameddine Kchir, Slim Haouet, Moncef Zitouna

**Affiliations:** 1 Pathology Department, La Rabta Hospital, Tunis, Tunisia; 2 Neurosurgery Department, La Rabta Hospital,Tunis, Tunisia; 3 Tunis El Manar University, Tunis, Tunisia

**Keywords:** Sarcoidosis, nervous system, pathology

## Abstract

Sarcoidosis is a multisystem disease of unknown cause and with a worldwide distribution. Involvement of the central nervous system occurs in a relatively small number of patients with sarcoidosis. Isolated neurosarcoidosis without signs of systemic disease however is a rare. In this report, we present an unusual case of neurosarcoidosis with intra cranial mass mimicking radiologically a glioma. Pathological examination revealed intraparenchymatous necrotising granulomatous lesions. After clinicopathological correlation, the diagnosis of a necrotizing cerebral granulomatosis (neurosarcoidosis) with atypical systemic involvement was made. Because of its non-specific clinical presentation and neuroradiological imaging characteristics, intracranial neurosarcoidosis remains a very difficult diagnosis, particularly in the absence of systemic signs of the disease.

## Introduction

Necrotizing granulomatous lesions of the central nervous system (CNS) are aetiologically diverse. These include chronic infections such as fungal or mycobacterial, as well as non-infectious causes such as Wegener granulomatosis, idiopathic pachymeningitis and neurosarcoidosis of the necrotizing sarcoid granulomatosis variant. An accurate diagnosis is critical to deciding the optimal treatment strategy, which can be diametrically opposite (intensive antimicrobial regimen vs immunosuppressants). Therefore a systematic thorough diagnostic work-up, clinicopathological correlation and appropriate follow-up are essential elements of patient management [1, 2].

## Patient and observation

A 32-year-old Tunisian woman presented with 2 years history of headaches. The patient's past medical history was no significant. She did not have a history of sinusitis, nasal deformity, or oral ulcers. she was not on any regular medications. Routine laboratory tests including renal function were normal. Chest x-ray showed neither lung lesions, nor mediastinal lymphadenopathy.

**Figure 1:**
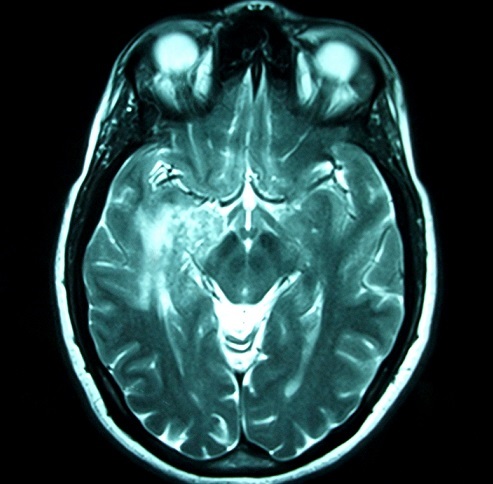
Cranial MRI: T1 post-contrast image in axial view: Enhancing parenchymal nodule involving the left temporal lobe, the lenticular nucleus and the internal capsule

**Figure 2:**
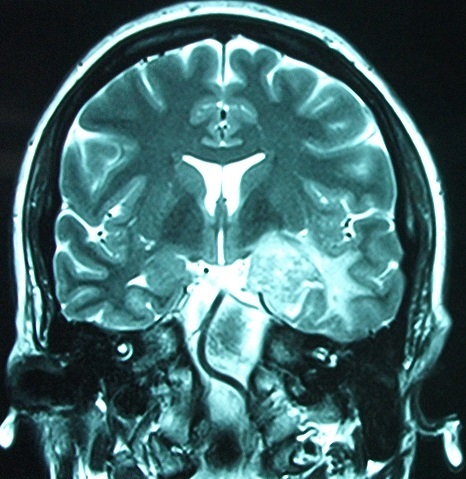
Cranial MRI: T2 image in coronal view: Nodule with surrounding edema

After an initial CT scan showed a left temporal lobe mass, he was referred to neurosurgery. A follow-up Magnetic resonance neuro-imaging studies (MRI), with and without contrast, identified a left temporal globular mass involving the lenticular nucleus and the internal capsule with edema and signal intensities suggestive of glial neoplasm (Figure 1, Figure 2). Administration of gadolinium resulted in significant enhancement in clods. The patient underwent a left subtemporal craniotomy with complete resection of the mass.

**Figure 3:**
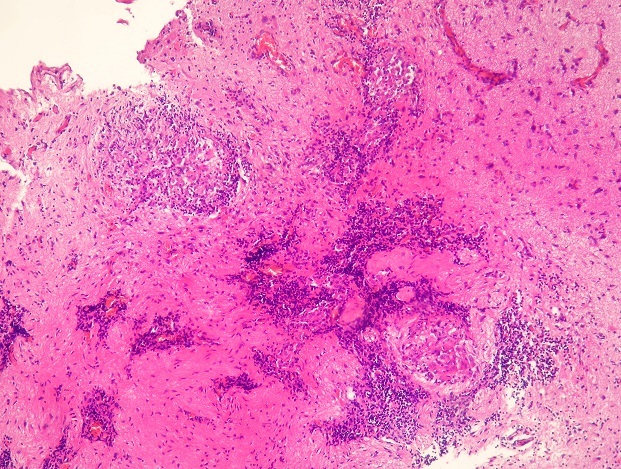
Granulomatous process involving neuroglial tissue (H&E x40)

**Figure 4:**
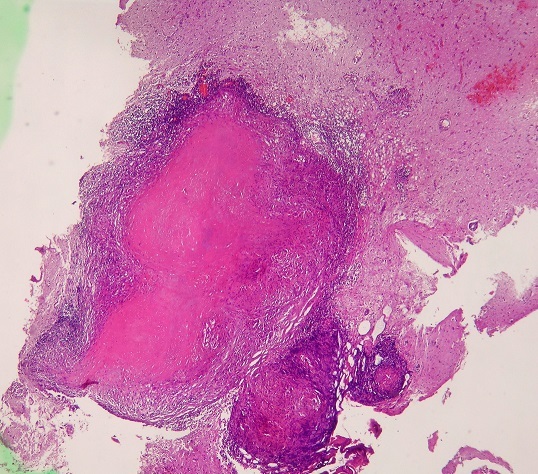
Nectrotising Granuloma with a central region of necrosis (H&E x 100)

**Figure 5:**
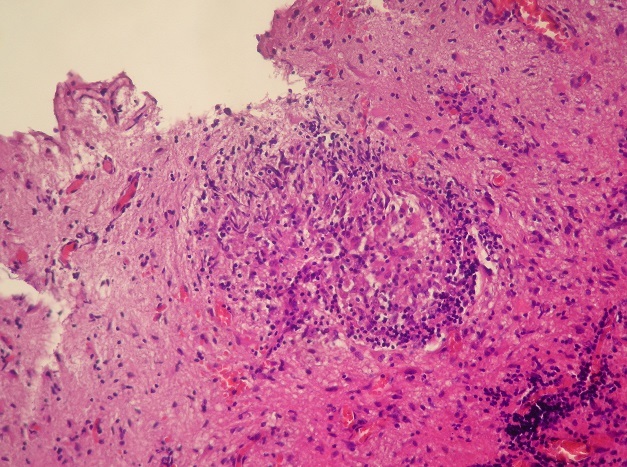
Granuloma with epithlioid cells and lymphocytes (H&E x200)

**Figure 6:**
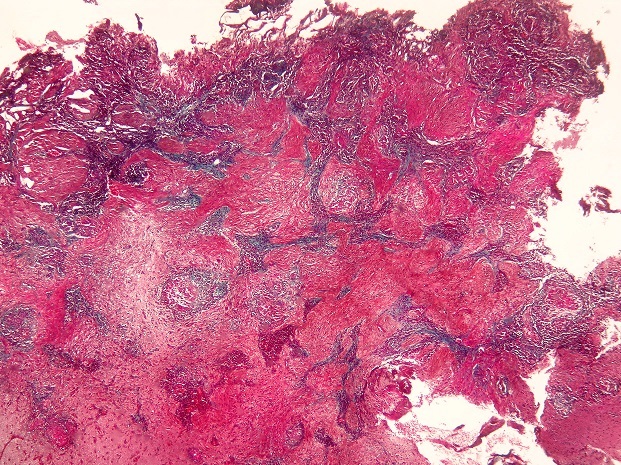
Masson trichrome staining highlighting Fibrosis surrouding granulomas (TM x 40)

Pathological examination revealed neuroglial tissue heavily involved with multiple granulomas (Figure 3). These lesions consisted of focal areas of fibrinoid necrosis, surrounded by a rim of mononuclear cells (Figure 4). The latter often had either oval-shaped or elongated irregular and indented nuclei. Most cells also had a generous amount of pink cytoplasm (epithelioid cells) (Figure 5). Intermixed with the epithelioid histiocytes were a modest number of lymphocytes and plasma cells, although acute inflammatory infiltrates including segmented neutrophils or eosinophils were not seen. Fibrosis surrouding granulomas was highlighted with Masson Trichrome staining (Figure 6). There was no definitive vasculitis, although some vessels had inflammatory cells around them. Sections were stained with periodic acid-Schiff and did not reveal any fungal organisms. Stain for mycobacteria (Zeihl-Neilsen) was negative.

Based upon the histopathology of the resected mass lesion, the two main differential diagnostic considerations were a mycobacterial infection (likely tuberculosis) and sarcoidosis. However, negative special stains, negative skin test for tuberculosis and normal Chest x-ray, nearly ruled out an infectious aetiology.

Postoperatively, the patient underwent corticotherapy. She had a partial motor deficiency of the right hemibody due to the achievement of the internal capsule. She is currently being retraining and she will have a fruther control.

## Discussion

Sarcoidosis is an idiopathic, complex, multisystem inflammatory disease of unknown aetiology, characterised by non-caseating [3]. It has been described in patients of all ages (usually under 50 years), sexes (more in women) and races. However, Americans and Africans are affected more often and tend to have a more severe and chronic disease, likely related to genetic associations within the human leukocyte antigen region [1, 4]. The clinical course is characterized by episodic exacerbations that are self-limited and may or may not respond to immunosuppressive therapy [2]. Classically, the lungs and mediastinal lymph nodes are involved, occurring in 90% of patients, although there often is variable involvement of other organ systems (eyes, skin, liver, spleen, salivary glands, heart, nervous system, muscles, and bones.) [5, 6]. Pathologic diagnosis relies heavily on the exclusion of other causes of non-necrotizing granulomata.

Sarcoidosis involving the central nervous system (CNS; neurosarcoidosis) affects only approximately 5-15% of patients with systemic sarcoid [2, 3]. It has been reported to involve all parts of the CNS, and its coverings including supratentorial and infratentorial compartments as well as the eye and the pituitary resulting in a wide spectrum of clinical syndromes [2, 5, 7].

The clinical features are protean. After cranial neuropathy (unilateral facial nerve palsy), headache is the most common manifestation of neurosarcoidosis, It has also been associated with ataxia, weakness, seizures and neuropsychiatric dysfunction, including dementia, amnesia, depression, delirium and psychosis [1, 2, 3].

Imaging studies are nonspecific, revealing a poorly defined mass with a variable degree of surrounding edema. Thus, in the appropriate clinical setting, neurosarcoid should be included in the differential diagnosis of a CNS mass [2, 8]. Magnetic resonance imaging is a very sensitive diagnostic tool for detecting intracranial abnormalities due to neurosarcoidosis. Parenchymal mass lesions or granulomas are a fairly common manifestation of neurosarcoidosis, with 35% of cases presenting as multiple supratentorial and/or infratentorial masses and 15% as solitary masses. These lesions are often intimately associated with leptomeningeal involvement and may represent centripetal spread of the disease. Intraparenchymal masses typically show enhancement as it was mentioned in our case. Initially, the lesions can be hypointense on T2,but are otherwise hyperintense. Central necrosis is rare. The MRI differential diagnosis includes particularly gliomas as in our case, metastatic disease, demyelinating disease and infarct.

Cranial nerves are affected in up to 50% of patients with neurosarcoidosis. There is poor correlation between imaging findings and clinical symptoms. While facial nerve deficits are most commonly found clinically, the optic nerve is the most common cranial nerve to appear abnormal on MRI.

Leptomeningeal involvement occurs in 40% and dural involvement in 34% of patients with neurosarcoidosis. The pituitary gland, infundibulum, or hypothalamus are affected in 18% of cases. Intramedullary involvement occurs in up to 25% patients [5, 9]. The cavernous sinus is rarely involved in neurosarcoidosis.

Although the wide spectrum of imaging findings in neurosarcoidosis is well documented, there is a paucity of data to show concordance between imaging findings and clinical information, the expected evolution of imaging abnormalities with immunosuppressive therapy, and the possible prognostic significance of imaging features [5].

Abnormal laboratory findings such as pancytopoenia and elevated serological findings such as C-reactive protein (CRP), erythrocyte sedimentation rate (ESR) and angiotensin-converting enzyme (ACE) levels, are all non-specific(BMJ2011). Lumbar puncture is useful in ruling out other neurological disorders, in particular infectious, but cerebrospinal fluid findings are not specific [7].

Despite the varied clinical and radiological appearances, the histopathologic hallmark of sarcoidosis is the presence of sarcoidal granulomas.

Sarcoidal granulomas, often described as “tight” granulomas, are discrete, round to oval, and composed of epithelioid histiocytes and multinucleate giant cells which may be of either Langhans or foreign body type. Generally, the type of multinucleate histiocyte present in a granuloma is not helpful in arriving at a specific histological diagnosis. Giant cells may contain asteroid bodies, conchoidal bodies (Schaumann bodies), or crystalline particles. Typical granulomas are surrounded by a sparse rim of lymphocytes and plasma cells, and only occasional lymphocytes are present within them. Consequently, they have been described as having a ‘naked’ appearance. Although the granulomas may be in close proximity to one another, their confluence is not commonly found. With reticulin stains, a network of reticulin fibers is seen surrounding and permeating the histiocytic cluster. The classic sarcoidal granuloma, compared with other types of granulomas, is relatively devoid of central necrosis (non-caseating granulomas). An accompanying granulomatous vasculitis may be apparent [10].

Necrotizing sarcoid granulomatosis, initially described by Liebow in 1973 (9), has been described even less frequently than its non necrotizing counterpart. Since that initial report, a few cases involving the central nervous system with histologic confirmation, have been reported in the literature [2].

The differential diagnosis of necrotizing granulomas includes chronic infections such as typical and atypical mycobacterium, fungi ( histoplasmosis, aspergillosis, cryptococcosis), and Wegener's granulomatosis, as well as foreign body type granulomatous inflammations. Therefore, a diagnosis of sarcoidosis, necrotizing or non-necrotizing, should be made only after other causes of granulomas have been excluded. Mycobacterial and fungal causes can be excluded by various histochemical stains and ideally by culture. Wegener's granulomatosis frequently has a characteristic clinical presentation in terms of both the clinical course and the distribution of lesions. Increased serum c-ANCA levels may further support it. Histologically, the granulomatous inflammatory process has a significant acute inflammatory component, accompanied by a large number of segmented neutrophils and a necrotizing vasculitis. The necrosis of Wegener's also often creates geographic patterns and may be surrounded by palisading histiocytes. NSG patients typically have a long clinical course with a significant, favorable response to steroids. These features clearly rule out infectious causes, particularly when supported by negative cultures [2, 6].

The management of neurosarcoidosis is not standardised. Immunosuppressive therapy may be interesting and should be initiated with corticosteroids. Other immunosuppressive drugs should be added in severe cases or after frequent recurrences. Its prognosis cannot be easily predicted. Natural outcome is poor.

However, receiving corticosteroid therapy lesions may regress partially or completely [1, 6, 7].

## Conclusion

The diagnosis of neurosarcoidosis remains in the purview of clinical suspicion. Unlike systemic sarcoidosis, there is difficulty in making tissue diagnosis when involvement of CNS is suspected. MRI studie is sensitive in the detection of CNS inflammation but lack specificity, making the ascertainment of neurosarcoidosis a clinical challenge. In addition the low prevalence of the disease makes clinical trials difficult and therapeutic decisions are likely to be made from careful reporting from case studies.
